# Still in search of a herbal medicine: Culprit is poor methodological quality of research

**DOI:** 10.4103/0253-7613.51351

**Published:** 2009-04

**Authors:** Jaykaran

**Affiliations:** Department of Pharmacology, Govt. Medical College and New Civil Hospital, Surat, Gujarat, India. E-mail: drjaykaran@yahoo.co.in

Sir,

I read the editorial “Still in search of a herbal medicine…” by Thatte.[1] She mentioned that “in spite of increasing research in herbal medicines. there is still no product, no drug, that has made a difference to therapeutics”. She mentioned various problems inherently associated with herbal research such as identification of plants, standardization, toxicological data etc. I believe something else we should not overlook is “methodological problems associated with the animal studies”. Studies show that animal studies are poor predictors of effects in humans.[2] Among 20 reviews examining clinical utility, animal models demonstrated the potential to contribute significantly toward the development of human clinical interventions in only two cases.[3] There are various types of methodological problems associated with these studies. Some of these are as follows:[2]

- Disparate animal species and strains.
- Different models for inducing illness or injury with varying similarity to the human condition.
- Variations in drug dosing schedules and regimen that are of uncertain relevance to the human condition.
- Variability in the way animals are selected for study.
- Methods of randomization.
- Choice of comparison therapy (none, placebo, vehicle).
- Reporting of loss to follow-up.
- Small experimental groups with inadequate power.
- Simplistic statistical analysis that does not account for potential confounding.
- Failure to follow intention to treat principles.
- Nuances in laboratory technique that may influence results may be neither recognized nor reported—e.g. methods for blinding investigators and selection of a variety of outcome measures, which may be disease surrogates or precursors and which are of uncertain relevance to the human clinical condition, etc.

I observed that in 2007 and 2008 among the articles published in IJP 84% were animal studies and many of the studies incorporated one of the problems mentioned above, such as randomization and blinding which can easily be done in animal experiments. Unfortunately either randomization or blinding are not done or are not declared in the many articles published in the journals.[2,3] In clinical research where the drug is administered to the human, both randomization and blinding are very important.[4,5] It has been shown that human clinical trials that lack randomization or blinding often overestimate the magnitude of treatment effects.[5] Animal studies that do not utilize randomization and blinding are more likely to report a difference between study groups than studies that employ these methods.[4] These methodological problems should be properly addressed while conducting research in animals.
